# The trap of a closed fist: Is democracy capable of preventing a global ecological catastrophe?

**DOI:** 10.1038/s44319-024-00341-0

**Published:** 2024-12-12

**Authors:** Vladimir Leksa

**Affiliations:** Laboratory of Molecular Immunology, Institute of Molecular Biology, Slovak Academy of Sciences, Bratislava, Slovakia

**Keywords:** Economics, Law & Politics, Evolution & Ecology, History & Philosophy of Science

## Abstract

Metaphors reforging science and art may help us to find a way to upgrade democracy so that it makes decisions with a positive impact for the next generations.

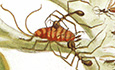

In May 2024, the seventh Starmus festival was held in Bratislava, Slovakia, under the theme “The Future of Our Home Planet”. More than 50 leading scientists from climate science, environmental science, astronomy, the natural sciences, and artificial intelligence, including nine Nobel Prize laureates, gave lectures, and well-known musicians performed, Jean Michel Jarre, among many others. Founded by the astrophysicists and musicians Brian May and Garik Israeli, Starmus, which combines the words Stars and Music, is a celebration of science and the arts. Once, these two domains of human activity belonged inseparably to each other (Fig. 1). That is why the unattainable work of Leonardo da Vinci, in which creative and analytical moments enhanced each other, never ceases to enchant us.

**Figure 1:**
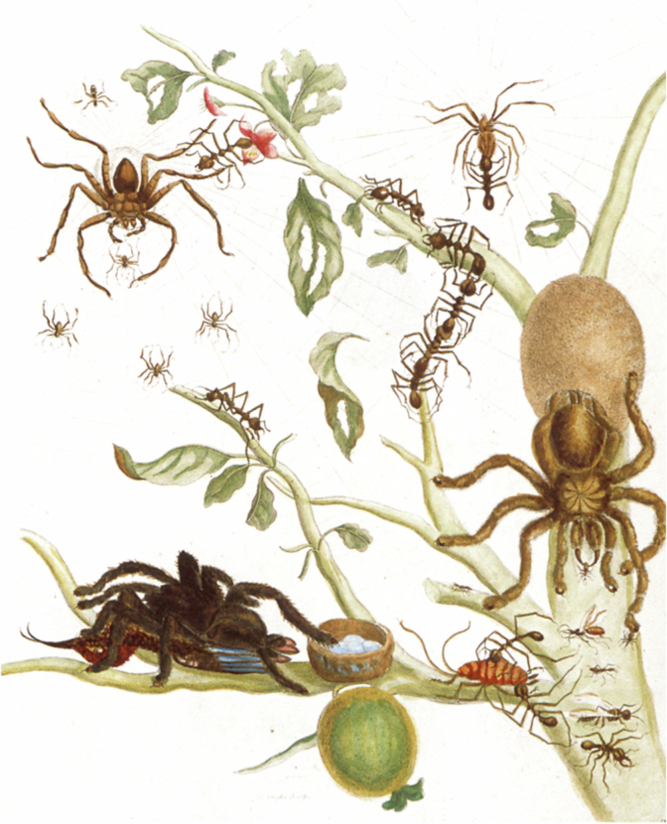
*Metamorphosis insectorum Surinamensium* “spiders, ants and hummingbird on a branch of guava” (1705) by Maria Sybilla Merian. Colored copper engraving. Wikimedia / Public Domain.

## May metaphor reforge again what was broken earlier

Sadly, these paths of creativity and discovery somehow diverged since da Vinci’s time. But science and art have always belonged together; it is knowledge what binds them. Through their work, artists report about themselves, their thoughts, experiences, feelings, and about something secret, subjective, hidden from others that does not have its own name. Scientists, on the other hand, investigate with impeccable precision the objective laws of the world, which universally apply to all of us. For this, it is necessary for artists to be true to themselves and for scientists to be true to reality.

“But science and art have always belonged together; it is knowledge what binds them.”

The main tool for creating a work of art and describing a scientific discovery is the metaphor. By means of a precise metaphor, artists let us know something about themselves and discover it in our own lives; with the help of a metaphor, scientists explain to us the principles of the universe, from atoms through cells to distant nebulae. When William Shakespeare wrote in his Sonnet XVIII: “So long as men can breathe or eyes can see, so long lives this, and this gives life to thee,” he did not intend to assess someone’s life expectancy, but to impress upon us that there are things around us that will be here long after us, that transcend us, and without which we could not live or love. Likewise, when Berthe Morisot painted her daughter Julie, she did not do it for a family album, but to catch the ephemeral reflection of light in her hair. And when Albert Einstein published E = mc^2^, he didn’t want to impress others with his calculation skills, but to express what matter is capable of, how it can be used or abused, and why it is good to have deep respect for it.

All of them used metaphors. And with the help of metaphors, it may be reforged again what was broken earlier: the imaginative and inquisitive hemispheres of the human mind, art, and science. Only then can we make our world a better place to live.

… “with the help of metaphors, it may be reforged again what was broken earlier: the imaginative and inquisitive hemispheres of the human mind, art, and science”.

## Humankind as the Titanic

At the Starmus festival, such a metaphor was conveyed by Steven Chu, a Nobel laureate in Physics and former Secretary of Energy under US President Barack Obama. Chu spoke about the climate crisis and how to avert a global environmental disaster. He compared our civilization with a great ocean liner heading towards an iceberg. Keeping course will inevitably result in a crash and disaster.

Hard-turning an ocean liner to avoid collision is not an easy task. It requires a complex series of maneuvers and an experienced and educated crew. Moreover, even if the crew performs well, it is not certain whether the ship will avoid the iceberg; owing to the enormous momentum and inertia of an ocean liner, it takes time and distance before the change, of course, is reflected in the resulting movement. Wherein lies the parallels with humankind? If all the countries of the world were to immediately stop greenhouse gas emissions today, it would take decades before a positive effect would set in. This means that any actions done by the current generation will be appreciated by the generation who grows up at the end of this century.

The metaphor likening today’s humanity to a ship in jeopardy should be clear enough to start taking the situation seriously and make a quick change of course. But at the same time, the whole thing is a little bit more complicated. In the upper well-lit salons, the orchestra keeps playing, passengers dance, eat caviar and crab mayonnaise and drink champagne. At the same time, a fire is raging on the upper deck, contagion is spreading on the middle deck, and the mechanics report leaking water in the lower decks. Moreover, there are rumors of a rapidly approaching iceberg. And the passengers have chosen as the ship’s officers from among themselves the most foolish ones who know nothing about the construction and dynamics of the ship, who have no idea about the existence of sea currents and storm zones, no clue how to use a rudder, to say nothing of the compass. Yet, their ignorance does not stop them from blatantly boasting of how they can fix everything, and that there is no reason to stop dancing and drinking champagne.

“The metaphor likening today’s humanity to a ship in jeopardy should be clear enough to start taking the situation seriously and make a quick change of course.”

## To be happy means to be free

In terms of an ocean liner’s operation and maintenance, the economically most developed countries, the ones that share the values of freedom and democracy, have the greatest authority. A lapidary question is, therefore—this time without metaphor—whether a system built on such values is capable of preventing a global ecological catastrophe. Those whose answer is negative, and there are not a few of them among younger people and adolescents, may easily convert their disappointment of liberal democracy into affection for autocratic regimes, hence, leave the control over the ship to a dictator.

This would be a tragic mistake. Dictatorships with their disrespect for life and nature will never solve the environmental crisis—just look at the environmental disasters that communism has left. But neither will democratically elected incompetent and uneducated populists. The only hope is to upgrade the democratic system so that it is able to face today’s challenges and make decisions that benefit the generations to come.

“The only hope is to upgrade the democratic system so that it is able to face today’s challenges and make decisions that benefit the generations to come.”

The modern democracy of the West is a legacy of the ancient *demos* of Athens. Pericles, the first strategos of Athens, in his famous funeral oration to honor the Athenians who died in the war with Sparta, summed up the essence of democracy: “To be happy means to be free” (Thucydides, History of the Peloponnesian War, Late 5th century BC). Across many centuries, this principle found its way into the preamble of the US Declaration of Independence along with other nations’ constitutions.

Modern science proves the notion that freedom is a basic prerequisite for happiness (Scholten et al, 2017). According to psychologists, free decision-making helps both children and adults to lead a full and successful life (Layland et al, 2018; Li et al, 2016). Free press contributes to a sense of happiness throughout society (Takahashi and Tandoc, 2016). In addition, the notion of free will significantly improves the communication skills of people with autism (Garcia-Villamisar and Dattilo, 2010). The same seems to apply to animals. Veterinarians have found that freely grazing cows have more offspring than those confined in cowsheds. But the former give less milk than the latter (Hovinen et al, 2009; Valde et al, 1997). This is understandable: to be happy means to be free. In the open, cows spend more time with social life, whereas in the cowshed, they can do little more than eating and lactating.

At the same time, with too much information available, the freedom to make your own choices becomes more challenging, even daunting, which does not necessarily make us happier. It is called choice overload (Misuraca et al, 2024). Moreover, people can be happy even in the most despotic conditions. According to the Austrian psychiatrist Viktor Frankl, many people can find bits of freedom and happiness even in the cruelest circumstances (Schimmoeller and Rothhaar, 2021). He realized this in the concentration camp where he had been imprisoned for years. Even there—and what can be more terrible than an extermination camp—people were able to experience a sense of intense personal happiness in moments that seem completely banal to us: a conversation with a fellow prisoner or a memory of their wife. Those moments gave them meaning and the will to live.

Thus, the relationship between freedom and happiness is not as straightforward as it might seem at first glance. We live in a democratic system. But are we happy? Are we really free? Do our lives have meaning?

## Error #2024 – System upgrade required

In India, farmers supposedly catch monkeys using a little trick. They make a small hole in a hollowed-out coconut and put grains of rice inside. A gluttonous monkey pops its hand in, grabs a handful of rice, but when it tries to take the hand out, it is not working. The closed fist does not pass through the opening. The animal is free to release the grains and pull its hand comfortably out; yet, it will not give up its prey. The hand is in spasm. The monkey, happy in the beginning, becomes an unhappy prey itself.

I am not sure if it really works that way, but I think, as a metaphor, it’s quite accurate. In humans, the closest relatives of apes, it works the same. We feel and we understand how the climate is changing, we see that plant and animal species are disappearing in front of our eyes, and in spite of this, we won’t do anything that would disrupt our consumption habits. We are trapped by a closed fist. It seems that life in a free and democratic society does not automatically make us free and happy. Thus, Pericles’ concept of happiness, freedom, and democracy should be revisited.

There are multiple levels of morality. The basic one concerns the well-being of oneself; the next level expands to the family, which is upper a ceiling, for example, for mafias members; next comes solidarity with human society; another, higher level represents engagement with nature; and the highest level of morality is feeling responsibility for the world that remains after us, that will be here even if we are long gone and forgotten. We should bear in mind this kind of morality and meaning when we upgrade democracy.

Liberal democracy stands on three pillars: legislative, executive, and judiciary. In other words, on an elected parliament, representative government and independent courts. However, it seems that these three pillars are no longer enough to ensure the stability of democracy. Democracy has always been a bit leaky, since it has always decided about the lives of those who have no right to vote.

“Democracy has always been a bit leaky, since it has always decided about the lives of those who have no right to vote.”

At the time of Pericles, not all citizens were allowed to vote. Slaves and women, although they had a greater degree of autonomy in Athens compared to other cities, were not allowed to participate in the elections. Modern democracy was not different. It wasn’t until 1870 that the 15th Amendment to the US Constitution gave black men the right to vote. And women, regardless of skin color, won the right to vote only during the 20th century. Hence, democracy has been updated several times. Nowadays, all residents of democratic countries can go to the polls without any restrictions. And yet, there are still two exceptions.

## The fourth pillar of democracy

First, there are children and adolescents. It is their future which is decided in elections and still, it is not theirs to participate. This poses a significant problem that young people may not reach the education level required to comprehend complex issues of today’s world, as uneducated politicians will not be interested in maintaining an efficient education system. In contrast, they are primarily interested in making voters as ignorant as possible because this increases their chance to be elected. If such politicians are elected repeatedly, more voters grow up, who are more prone to believe any nonsense. The unavoidable outcome of this trend is that an autocrat will eventually come to power, who will completely cancel free elections.

Furthermore, polar bears, tigers, or codfish have no right to vote either, nor do maples, violets, or mushrooms. In one word, nature. It remains under firm control of human governments. Sad to say, nature itself will never win the right to vote. It is we who must vote on behalf of the environment. Certainly, many people in the world are perfectly aware of this responsibility; yet, it is questionable whether there are enough of them, whether the majority of legitimate voters are able to think in this way, because democracy, as Pericles had it long ago, means that “the administration is in the hands of the many and not of the few” (Thucydides, History of the Peloponnesian War, Late 5th century BC).

“It is we who must vote on behalf of the environment.”

We, therefore, need a fourth pillar of democracy: an independent system of education and environmental protection, which would not be under the control of politicians hatched from national elections, but instead managed by an international team of experts. In his essay “Divided we stand, united we fall!”, the philosopher Slavoj Žižek deduced the critical need of such a controlling body for democracy maintenance.

Immediately, this raises the question, who are to be the members of such a “council of the wise”? And who will assign them to the council? Or could we entrust the UN or the EU to manage both the educational system and environmental protection councils, given that either or not free from the influence of member states? Or should it be academic committees akin to the Nobel Prize selection or national academies of science? What about expanding the scope of the WHO? And then the third issue is: will national governments respect such an organ and implement its guidelines? And even if most democratic countries were to accept a fourth pillar, how should we convince autocratic societies to adhere to their counsel? Questions and questions; nonetheless, we have to start somehow. It is akin to creating a genuine metaphor in art and science: we have to be true to ourselves and to reality. I am confident that governments, and corporations can do it, given that being true will be somehow beneficial to them. But a short-term advantage must be transformed into a long-term benefit for all.

Indeed, there are examples where governments have found common ground and agreed to cooperate for the benefit of the many. At any moment, thousands of planes from hundreds of airlines from countries with vastly different political regimes cross the airspace without colliding. They all take off and land safely at their destinations. This truly amazing feat is due to the International Civil Aviation Organization that controls and oversees global air traffic.

Another example is the Arctic Council, an intergovernmental forum of the Arctic border states to protect the Arctic environment that has achieved a high level of agreement despite the politically and economically divergent interests of its member states. Though enforcement is still a problem, there is also remarkable cooperation. On a global level, the Law of the Sea Treaty regulates all marine activities for 169 UN member states and the EU. Again, while it lacks enforcement in wide areas, the fact that so many nations have agreed to it is a remarkable political success.

Maybe the best illustration of mankind’s ability to cooperate in times of crisis is the elimination of freons (chlorofluorocarbons; CFCs), refrigerants that threaten the protective ozone layer in the stratosphere. Barely 30 years have passed from the first observations that CFCs deplete atmospheric ozone and their verification by scientists to an international consensus and policies that effectively ban the production and use of ozone-depleting chemicals (Andersen et al, 2013).

Having successfully resolved those crises, we are certainly capable of managing the global climate crisis too. But it demands an experienced and educated crew. It demands focusing not just on the present moment, but on the time to come after us, like in Steven Chu’s metaphor of the ship heading towards an iceberg. Just like turning an ocean liner around, making a fundamental upgrade of our democratic system to preserve our environment and our societies for the future is not an easy errand. After all, even the Starmus festival, mentioned at the beginning, opened with a spectacular, energy-consuming light show with lasers and drones. As long as we like it. It is a tough proposition to give up things that we like and have become accustomed to. It will be difficult to be true to ourselves and to reality. Difficult but not impossible.

“Just like turning an ocean liner around, making a fundamental upgrade of our democratic system to preserve our environment and our societies for the future is not an easy errand.”

## Supplementary information

Peer Review File
