# Supplementary material for: The trap of a closed fist: Is democracy capable of preventing a global ecological catastrophe?
Source: EMBO Rep. 2024 Dec 12;26(1):5–8. doi: 10.1038/s44319-024-00341-0 (PMC11723946; doi:10.1038/s44319-024-00341-0)
Supplement: Supplementary file 1 — Peer Review File [file 44319_2024_341_MOESM1_ESM.pdf]

EMBO reports

TITLE: The trap of a closed fist

Vladimir Leksa

Corresponding author(s): Vladimir Leksa (Vladimir.Leksa@savba.sk)

Review Timeline:

	Submission Date:	12th Nov 24
	Accepted:	26th Nov 24

Editor: Holger Breithaupt

Transaction Report: This article was editorially reviewed.
